# Disseminated *Mycobacterium avium *complex infection in an immunocompetent pregnant woman

**DOI:** 10.1186/1471-2334-6-154

**Published:** 2006-10-22

**Authors:** Joon Young Song, Cheong Won Park, Sae Yoon Kee, Won Seok Choi, Eun Young Kang, Jang Wook Sohn, Woo Joo Kim, Min Ja Kim, Hee Jin Cheong

**Affiliations:** 1 Division of Infectious Diseases, Department of Internal Medicine, Korea University College of Medicine, Seoul, Korea; 2 Department of Diagnostic Radiology, Korea University College of Medicine, Seoul, Korea

## Abstract

**Background:**

Disseminated mycobacterium avium complex (MAC) occurs mainly in immunocompromised hosts, which is associated with abnormal cellular immunity.

**Case presentation:**

A 26-year-old pregnant woman presented with fever and general weakness. Miliary lung nodules were noted on chest X-ray. Under the impression of miliary tuberculosis, anti-tuberculosis medication was administered. However, the patient was not improved. Further work-up demonstrated MAC in the sputum and placenta. The patient was treated successfully with clarithromycin-based combination regimen.

**Conclusion:**

This appears to be the first case of disseminated MAC in an otherwise healthy pregnant woman. Clinicians should be alert for the diagnosis of MAC infection in diverse clinical conditions.

## Background

*Mycobacterium avium *complex (MAC) causes three major clinical disease entities: pulmonary disease, disseminated MAC disease, and cervical lymphadenitis [1,2].

MAC has been found to colonize natural water, soil, indoor water systems, and hot tubs, and it can be isolated from the sputum of apparently healthy individuals [3,4]. Clinically, MAC pulmonary disease is more frequently seen in patients with underlying lung disease such as bronchiectasis, pneumoconiosis, and prior tuberclulosis, but approximately 24 to 59 percent of them occur in immunocompetent persons without evident predisposing factors [2,5].

In comparison, disseminated MAC disease has been mainly noted in immunocompromised hosts with impaired cellular immunity [1]. Recently, we experienced an interesting case of disseminated MAC disease, which occurred in an otherwise healthy pregnant woman. Pregnancy might affect on the patient's immune status. Herein, we report the unusual case with the review of previously published literatures.

## Case presentation

**Figure 1:**
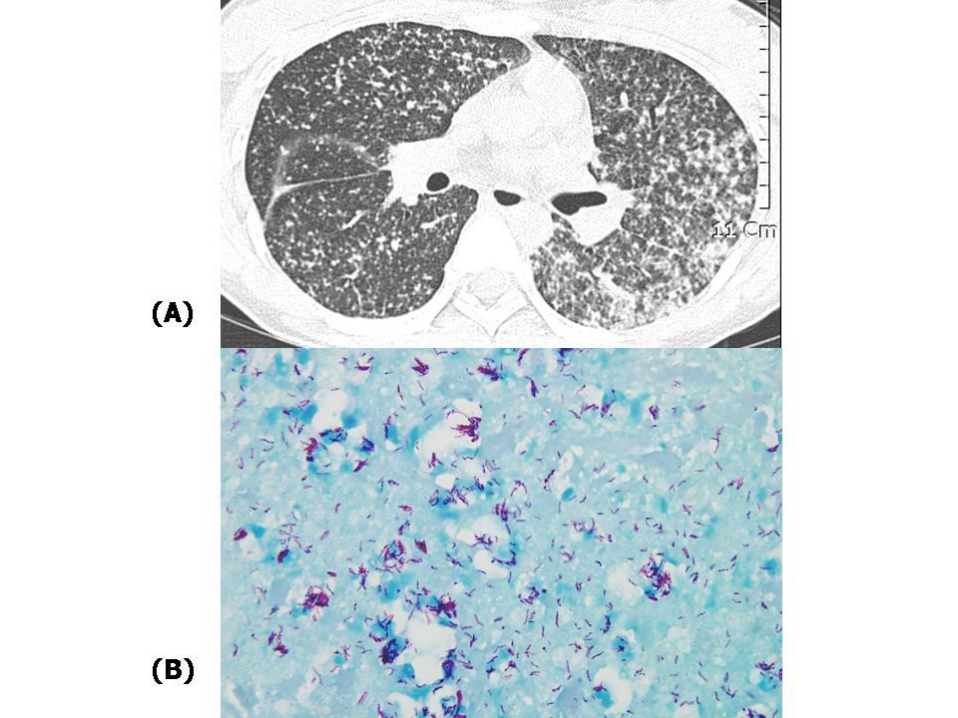
(A) High resolution computed tomography of lung shows multiple tiny nodules in the entire lung fields and conglomerated lesions in left lung fields. (B) Many acid fast bacilli were shown on Ziehl-Neelson stain of placenta tissue from the patient.

A 26-year-old pregnant woman presented to the Division of Infectious Diseases at Korea university Guro hospital, Seoul, Republic of Korea (ROK) with history of fever and abdominal pain for a week. She was a healthy hepatitis B virus (HBV) carrier that was vertically transmitted from her mother. The patient was in the 13^th ^week of her first pregnancy, and she did not take any medicine at all. The patient was admitted for work-up of her febrile condition. On admission, her vital signs were body temperature 38.1°C, blood pressure 100/60 mmHg, and pulse rate 86 times/minute. She complained of generalized weakness, loose stool, night sweating, and dry cough. On physical examination, mild right upper quadrant tenderness and decreased breathing sound on the right lower lung field were noted. Initial laboratory investigations showed mild anemia, with a hemoglobin level of 10.9 g/dL and a platelet count of 232 × 10^3 ^platelets/mm^3^. White blood cell (WBC) count was not elevated with 6,100 cells/mm^3^. Aminotransferase levels were elevated with levels of 127/117 IU/L in AST/ALT respectively. Alkaline phosphatase level (207 IU/L) was also increased. Serologic tests for HIV, Hepatitis C virus (HCV) and hepatitis A virus (HAV) were negative. Activity of HBV was not increased: hepatitis e antigen (-), hepatitis e antibody (+), HBV DNA (1.0 pg/mL). The patient's chest X-ray showed numerous tiny nodular opacities on both lungs, and high-resolution computed tomography of the chest demonstrated numerous randomly distributed nodules (Fig. 1A). Tuberculin test showed anergy. Those findings were compatible with miliary tuberculosis.

In ROK, tuberculosis is still highly prevalent, so the patient was initially suspected of having miliary tuberculosis in the lung. Anti-tuberculosis medication was started with isoniazid (300 mg qd), rifampin (600 mg qd), ethambutol (1.2 g qd), and pyrazinamide (1.5 g qd). Aminotransferase levels were not elevated further during the course of anti-tuberculosis medication. Sputum induction with hypertonic saline was performed because the patient only had intermittent dry cough and scanty sputum. The sputum study was negative for Mycobacterium on Ziehl-Neelson stain.

High fever persisted despite anti-tuberculosis medication. On day 15 of anti-tuberculosis medication, the patient's condition was aggravated; she suffered from severe dyspnea and hyperemesis. Fetal distress was suspected, forcing us to terminate the pregnancy. Pathologic specimens obtained from the placenta demonstrated many acid fast bacilli in Ziehl-Neelson stain (Fig. 1B). However, the patient was still febrile despite the termination of pregnancy and continued anti-tuberculosis medication. The authors believed that an immunologic response might have been responsible for the high fever and added oral steroid (oral prednisone; 1 mg/kg) to the patient's medication. The patient felt better and was no longer febrile. The patient was subsequently discharged from the hospital with those medications.

During outpatient follow-up, the patient complained of frequent episodes of fever and exertional dyspnea even though she was taking anti-tuberculosis medication. Chest X-ray revealed that the pulmonary disease worsened with multiple nodules in the entire lung. On day 45 of anti-tuberculosis medication, results from the initial series of sputum mycobacterial cultures on ogawa medium were available; Mycobacterium other than tuberculosis (MOTT) was demonstrated repeatedly. Result from placenta was same also. The patient's chest X-ray showed no improvement since discharge from the hospital, with persistent multiple tiny nodules in both lung fields. The medication was changed to clarithromycin (500 mg bid), rifampin (600 mg qd), ofloxacin (300 mg bid), and ethambutol (1.2 g qd).

**Figure 2:**
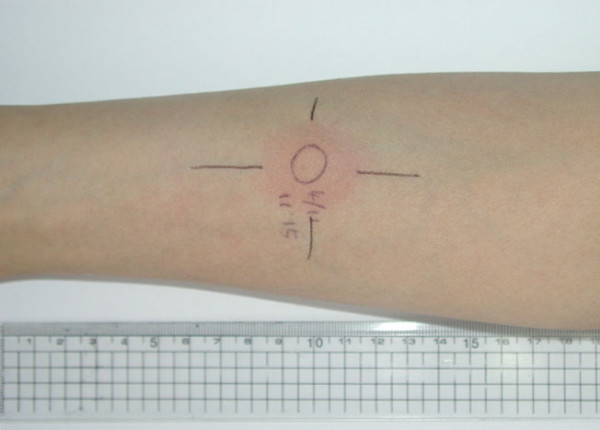
Tuberculin test was performed after the patient was stabilized with anti-MOTT (mycobacterium other than tuberculosis) medication; 2.5 cm sized induration was observed 48–72 hours later.

On day 60 of anti-MOTT medication, all the patient's symptoms were dramatically improved. Chest X-ray was nearly cleared up, and liver function tests resolved completely also. On day 105 of anti-MOTT medication, mycobacterial speciation culture report from sputum and placenta specimens revealed *Mycobacterium avium intracellurare *with isoniazid, ethambutol, and Para-aminosalicylic acid resistance. The drug regimen was adjusted in accordance with results from the antibiotic susceptibility test: clarithromycin, rifampin, and ofloxacin. The patient is on the 10 months of the new drug regimen with continuous improvement of her condition. Follow-up HIV serology was negative and the result of tuberculin test was converted to strong positive (2.5 cm sized induration), which were performed after 10 month medication (Fig. 2). No specific abnormality was noted on gynecologic examination, endoscopy, breast and abdominal sonogram.

## Discussion

We report a rare disseminated MAC case in an otherwise healthy, pregnant woman; there had been only sporadic case reports of disseminated MAC disease among immunocompetent adults [6-10]. Though the patient in this case was a HBV carrier, there was no evidence of liver cirrhosis on physical examination and abdominal sonogram. Platelet count was also normal. Repeated HIV serology was negative and tuberculin test showed strong positive reflecting normal cell-mediated immunity. Studies of malignancy work-up showed no abnormal finding.

Disseminated MAC disease is typically associated with defective cellular immunity such as hairy cell leukemia, chronic myelogenous leukemia and AIDS [11]. Tumor necrosis factor (TNF)-α and IFN-γ are both important in the defense against mycobacterial disease. Schematically, stimulated CD4^+ ^T-lymphocytes produce IL-12 and IFN-γ. These cytokines act on lymphocytes and macrophages to stimulate the production of IL-2, TNF-α, and reactive oxidative intermediates such as superoxide and nitric oxide that participate in the host defense against mycobacteria [12]. Patients with lymphoreticular malignancies or AIDS are at risk of disseminated MAC infection, usually when the CD4^+ ^count is <50 μL. In those patients, macrophages are unable to kill the intracellular mycobacteria largely because they lack the help of CD4^+ ^T-cells secreting INF-γ and TNF-α; these cytokines are necessary for the activation of macrophages.

Alterations of the immune status in the pregnant woman are necessary to allow mothers to tolerate genetically different fetal tissues during pregnancy. These alterations lead to impaired cell-mediated immunity with increased susceptibility to certain infections such as tuberculosis [13,14]. During pregnancy, the maternal immune system also shows a relative bias toward T helper (type 2) immunity. Therefore, we presumed that disseminated MAC infection in this case might be related to pregnancy state, even though immune-suppressed disease did not exist. Likewise, cytomegalovirus infection and disseminated aspergillosis had been reported as opportunistic infections in the pregnant women [15,16].

Since the mid 1990s, genetic abnormalities are known to be predisposed to infection by intracellular pathogens including MAC and other mycobacteria; mutations in the interferon γ receptor 1 (IFN-γR1) gene and natural resistance-associated macrophage protein (*nramp*) gene have been reported [5]. This case however, was not analyzed for these genes because no other family member revealed mycobacterial disease.

As for the diagnosis of disseminated MAC infection, though blood culture was not taken, positive cultures were identified from more than one organ (lung and placenta), and the patient showed compatible clinical/laboratory findings. Multi-drug therapy including clarithromycin or azithromycin is recommended for the treatment of disseminated MAC patients with AIDS based on available data [17,18]. In contrast, no standard treatment has been established for HIV-negative patients with disseminated MAC infection. In this case, we treated the patient with combination of rifampin, ofloxacin and clarithromycin, and it was effective. We excluded ethambutol from the treatment regimen according to the in vitro susceptibility test. Actually, however, the susceptibility test of MAC has no value in guiding the selection of drugs other than clarithromycin [19].

In this study, CD4 T cell count was not checked during follow-up period, which was the limitation of this report. However, the patient was previously healthy and WBC count was within normal range. The positive conversion of tuberculin test was checked during treatment period after therapeutic abortion, therefore we thought those could be an indirect evidence of intact cellular immunity.

In conclusion, this case highlights a series of problems associated with MAC disease including delay in diagnosis, undefined mode of transmission, potential severity of the disease, and difficulty in treatment. Clinicians should be alert for the diagnosis of MAC infection in diverse clinical conditions. Large-scale study is needed in those patients with disseminated MAC disease, who are immunocompetent and free from underlying lung disease: treatment regimens, treatment duration, virulence factors of MAC, and host immunity.

## Conclusion

We report a rare disseminated MAC case in an otherwise healthy, pregnant woman. The patient was treated successfully with combination of rifampin, ofloxacin and clarithromycin. Clinicians should be alert for the diagnosis of MAC infection in diverse clinical conditions.

## Competing interests

The author(s) declare that they have no competing interests.

## Authors' contributions

WSC, SYK, CWP, WJK, and MJK advised on the management of the patient and assisted in editing the manuscript. EYK performed the chest X-ray and CT reading. JYS and HJC drafted the manuscript and were involved in patient management and follow-up. All authors read and approved the final manuscript.

## Pre-publication history

The pre-publication history for this paper can be accessed here:


